# Ethics of Artificial Intelligence in Medicine

**DOI:** 10.7759/cureus.83567

**Published:** 2025-05-06

**Authors:** Min Kyu Park, Neil Ashwood, Neil Capes

**Affiliations:** 1 Trauma and Orthopaedics, University of Leicester, Leicester, GBR; 2 Trauma and Orthopaedics, University Hospitals of Derby and Burton NHS Foundation Trust, Derby, GBR; 3 Trauma and Orthopaedics, University of Wolverhampton, Wolverhampton, GBR

**Keywords:** artificial intelligence (ai), artificial intelligence in medicine, ethics and professionalism, health autonomy, medical codes of ethics

## Abstract

Artificial intelligence (AI) has shown great promise in becoming an integral part of healthcare, offering advancements in diagnostic accuracy, surgical precision, and personalised patient care in numerous medical specialties, including radiology and surgery. This paper explores the ethical implications of AI in medicine, with emphasis on the four key ethical principles of autonomy, beneficence, non-maleficence, and justice. The ethical challenges include concerns about patient consent, data privacy, clinical transparency, and the potential for AI to exacerbate health disparities. This paper explores the need for clear ethical guidelines and regulatory frameworks to ensure AI is used in a way that enhances healthcare without compromising ethical standards. As technology continues to advance, it is crucial to balance technological advancement with the fundamental principles of medical ethics to ensure that healthcare is delivered in a safe and compassionate manner.

## Editorial

Artificial intelligence (AI) is the study of expert systems that use the capability of computers to process different types of algorithms and models to perform cognitive functions such as problem-solving [1,2]. It was first described by John McCarthy in 1956, defining it as "the science and engineering of intelligent machines" [3], but it is believed that Alan Turing described the possibility of machines to simulate human behaviour [4]. AI comes in many different forms, ranging from machine learning from processing data to the use of robotics.

AI was first introduced to the medical field through the causal associational network (CASNET). CASNET was first used for consultations of patients with glaucoma in 1976 [5]. It used a network of disease knowledge and connected them into causal and associational relationships, the first system to ever do so. Therefore, it was able to suggest possible diagnoses, along with an explanation of the reasoning behind the diagnosis [6]. It laid the foundation for structured medical knowledge representation. Now, AI has deep roots within medicine, from booking appointments through Babylon, all the way to being used in radiology for quicker image recognition, to the everyday wearables such as Fitbit and Apple.

However, as the use of AI becomes more common, there are concerns being raised about the ethical aspects of AI use in medical practice. This article plans to identify and analyse some key ethical dilemmas that arise from the use of AI in healthcare.

AI in medicine

AI is first introduced into the careers of doctors early on, during medical school. When anatomy is being studied, a variety of tools are used to help students visualise the anatomical structures of the human body, ranging from cadavers to apps that use AI in order to show the human body in 3D. Medical research is a field where AI is also becoming increasingly used, especially in clinical trials where medical imaging is prominent. AI systems are beneficial to medical image processing by not only being able to automate tasks like tissue segmentation but also reducing human-induced variability that may cause biased results [7].

AI is also becoming more integrated in surgery, where robotic-assisted surgery is becoming more and more prevalent. The integration of AI into robots such as the Smart Tissue Autonomous Robot (STAR) allows small tasks such as suturing in anastomoses to become more accurate and efficient [8]. Many studies have shown that the incorporation of AI into surgical specialties has not only increased the accuracy in the diagnosis of certain conditions but also helped in assessing prognosis and potential post-surgical complications [9].

The specialty with the most AI being incorporated is radiology. There are AI systems where AI helps enhance the visualisation of various pathologies [10] and with the processing of the images, which enables faster diagnosis to be made [11]. Various examples include the Picture Archiving and Communication System (PACS), which has a system where automatic detection of liver, lung, cardiovascular, and bone disease is possible, and there have been cases where deep learning has been introduced to detect calcium in the coronary arteries to predict cardiovascular events [12]. However, AI algorithms rely on precise and comprehensive data that represent various patient demographics. If restricted data is used, it could lead to representation bias, reducing the efficiency and accuracy of AI in radiology [13].

Ethical principles

As described above, AI is being extensively used in healthcare with many benefits. However, there are certain ethical concerns that need to be considered and addressed. There are four main pillars when it comes to medical ethics: autonomy, beneficence, non-maleficence, and justice. The ethical dilemmas that accompany the use of AI will be discussed with an analysis regarding each pillar.

Autonomy

Autonomy is the principle that everyone has the power to make rational decisions and moral choices. Every patient who has demonstrated capacity can determine what treatment they can consent to or not.

When AI is being used in healthcare, patients should be informed about how AI is used in their care, including what data is collected, how it's used, and who has access to it. Consent should be explicit, especially for sensitive applications like genetic data analysis, so that patients are able to make informed decisions on what care they receive.

Patients should have control over their personal health data, including the right to access, modify, or delete their information from AI systems. The Data Protection Act (DPA) 2018 in the United Kingdom is an example of a legal framework that would ensure this happens, requiring that the collection and processing of personal data is fair, lawful, and transparent. Access to the data AI systems use about them, along with the ability to correct inaccuracies, should be given to patients, ensuring they are active participants in their care. Studies have been done to investigate suicide prediction tools using AI, using machine learning from data from electronic medical records (EMRs) and hospital records to assess suicide risk [14]. However, these raise the question of whether the patients would be notified on how their data is being used.

This also brings up the question of data privacy and security. When AI collects, stores, and shares patient data, there is always the risk of data breaches. Considerations should be made to think about what standards should be put in place when these data breaches do occur.

Beneficence

Beneficence explains the role of the doctor to act in the best interest of the patient.

AI should be developed to genuinely improve patient health outcomes, such as through more accurate diagnoses, personalised medicine, or better management of chronic conditions. Currently, AI-incorporated watches, such as the Apple Watch, are able to detect cardiac abnormalities such as arrhythmias. This ability allows patients with arrhythmias to be detected much earlier.

Medical decisions influenced by AI should be transparent. Patients and clinicians should understand how AI comes to the decisions that are made. Systems where AI can provide explanations for their decisions in a way that is understandable to patients and healthcare providers need to be implemented. This could involve natural language explanations or visual aids showing how a decision was reached. One such example is the case of IBM Watson, an AI-driven tool that was invested in to aid cancer treatment in oncology. The AI decision-making process was not transparent, being known as a black-box system. In this way, healthcare workers would be able to ensure that the AI systems are working for the benefit of the patient.

Non-maleficence

The principle of non-maleficence is the obligation of healthcare workers not to harm patients. This includes not to kill or cause pain or suffering to patients.

There should be mechanisms to audit AI systems for performance, bias, and ethical compliance. There should be systems in place for AI to learn and adapt in an ethical manner, with human oversight to ensure ethical standards are not compromised as AI evolves. Along with these audits, rigorous testing and validation of AI systems are necessary to ensure they are safe and effective for use in clinical settings. Strong safeguards are needed to protect patient data from breaches, misuse, or unintended exposure.

Furthermore, in order to ensure that no harm is done to patients, it is vital that healthcare workers use AI to support decisions to be made. In the example of the case of IBM Watson, there was a mismatch between Watson's recommendations and the clinical assessments of clinicians. Therefore, there was a discrepancy between the two assessments, with the clinicians losing trust in the AI system. A human-in-the-loop model could be applied to prevent this problem from occurring. If AI potentially makes decisions that may harm the patient, clinicians should be able to override these decisions. It would actively allow humans to give direct feedback to the algorithm, with the aim of enhancing the accuracy, reliability, and adaptability of these AI systems. Not only would this reduce the harm done to patients, but it would prevent over-reliance on AI.

Justice

Finally, justice explains how the treatment of patients must be fair, equitable, and appropriate.

There is the possibility that the widespread incorporation of AI into healthcare could lead to discrimination. The discrimination may not be as obvious in countries where healthcare is run by government-run bodies, such as the National Health Service (NHS) in the United Kingdom. However, it could lead to a greater disparity between socioeconomic groups where healthcare is private. It is most likely that treatment that involves new technologies, such as the incorporation of AI systems, would be very costly. Thus, it would only be available to certain groups, leaving the poor and vulnerable unable to afford the treatment. For example, there are cases in the United States where healthcare professionals are aware that certain treatments would not be available to the majority of the population [15]. Discrimination would occur not only between the public but also between low- and middle-income countries. Wealthier countries would be able to afford the new technological advances, leaving the low-income countries without the availability of AI systems. It needs to be ensured that the benefits of AI in healthcare are accessible to all segments of the population, not just those who can afford advanced medical technologies.

AI should not contribute to systemic biases; for instance, algorithms should not favour one group over another based on socioeconomic status, race, or gender. If this is not tackled, it may amplify disparity amongst different races, genders, and socioeconomic statuses. There was a report by Obermeyer et al. that found evidence of racial bias in a widely used algorithm in the United States, where Black patients were consistently assigned a lower risk score than White patients with the same condition by the algorithm [16]. AI systems must be designed to avoid perpetuating or exacerbating existing health disparities. This includes addressing biases in data sets or algorithms that could lead to discriminatory practices.

AI systems should be continuously evaluated for biases in data sets, algorithms, and outcomes. This would involve using diverse data sets from various demographics to prevent AI from perpetuating existing disparities in healthcare. In order to do this, diverse perspectives from different teams, including clinicians, ethicists, and community representatives, are needed to identify and mitigate biases at the design stage.

Responsibility

There must be clear lines of accountability when AI is involved in healthcare decisions. This includes who is responsible if an AI system leads to harm or when errors occur. For example, if AI makes errors such as mechanical failures in the operating room that may result in death, would the AI be held legally responsible? Legal frameworks need to evolve to address issues like liability in cases where AI contributes to medical errors or fails to perform as expected.

Patient-doctor relationship

Healthcare providers need education and guidelines on how to integrate AI into their practice ethically, ensuring AI supports rather than supplants human judgment and empathy. Along with this, healthcare workers need to be educated on how to effectively collaborate with AI, understanding its capabilities and limitations, to enhance rather than undermine the therapeutic relationship. The roles of AI need to be clearly defined. It needs to be ensured that AI supports human judgment. AI can handle routine tasks, data analysis, or preliminary diagnostics, freeing clinicians to focus on complex decision-making and patient interaction.

Furthermore, empathy is an integral part of holistic and patient-centred medicine, being able to understand, share, and respond appropriately to the emotions and experiences of patients. It is a main part of the development of the patient-doctor relationship. The introduction of AI could possibly enhance this with the reduction of the clinician's workload, allowing them to focus and spend more time engaging with patients. A study by Kerasidou has explained that AI algorithms would be able to free up the time of the healthcare staff, which could in turn lead to increased empathy and compassion from the staff [17].

However, the over-reliance on AI could lead to the opposite, making it vital to educate healthcare workers on the importance of personalised and compassionate care and the importance of empathy in medicine.

Guidelines for AI

Guidelines are needed from governments, medical institutions, and technology companies that regulate AI in medicine. As healthcare is a global concern, international collaboration is crucial to set ethical standards for AI use in medicine. International or national guidelines that specifically address AI in healthcare, covering aspects like patient consent, data privacy, and accountability for AI decisions, are needed. As AI technology evolves, so too should the legislation. This might mean creating frameworks that are flexible enough to adapt to new technological advancements without compromising ethical standards. These ethical concerns have been acknowledged by the World Health Organization (WHO), which has released guidance and recommendations regarding the use of AI in health. The WHO has recommended assigning regulatory agencies to assess and approve the use of AI, along with using laws, policies, and regulations to ensure these algorithms meet ethical obligations. It has also been recommended to have mandatory post-release auditing and impact assessments [18].

Development and deployment of AI in healthcare should be monitored by ethical review boards or similar bodies. These ethical review boards, which should include not just technologists but also ethicists, patient advocates, and legal experts, need to be established specifically for AI applications in healthcare.

It is a known fact that technology is constantly evolving and medical technology is no exception. It is inevitable that further advancements in technology will bring new ethical dilemmas that need to be tackled. Therefore, it is integral to balance new technology with the ethical side of medicine. By focusing on these areas, AI can be seamlessly integrated into healthcare systems in a way that enhances patient care, supports healthcare providers, and maintains or even elevates the human aspect of medical practice. This approach not only respects the ethical dimensions of healthcare but will also leverage AI to make healthcare more personalised, efficient, and accessible.
